# Adamantinoma-Like Ewing's Family Tumor of the Sino Nasal Region: A Case Report and a Brief Review of Literature

**DOI:** 10.1155/2019/5158182

**Published:** 2019-05-07

**Authors:** Pushpa Mahadevan, Subramaniam Ramkumar, V. P. Gangadharan

**Affiliations:** ^1^VPS Lakeshore Hospital, Nettoor, Maradu, Ernakulam, Kerala 682040, India; ^2^Neuberg Anand Reference Laboratories, Neuberg Diagnostics Pvt. Ltd., Thombra Arcade, Elamakkara Road, Kaloor, Cochin-682017, India

## Abstract

Ewing's sarcoma family of tumors (EFTs) are malignant mesenchymal tumors with a predilection for bone and soft tissue. They are characterized by their monomorphic small blue round cell morphology. However rare morphologic variants of EFTs can also show overt epithelial differentiation in the form of squamoid differentiation along with strong cytokeratin expression. This particular subset of EFTs are known as adamantinoma-like EFTs which can be difficult to differentiate from epithelial head and neck malignancies. Here we report a case of sinonasal adamantinoma-like EFT in an 18-year-old male patient. The lesion differed from a typical EFT by means of overt squamoid differentiation which showed a basaloid appearance with peripheral palisading. The immunohistochemistry was positive for pan-cytokeratin, p40, p63, ERG, FLI1, and CK5/6. It was negative for actin, desmin, and WT-1. Initial diagnosis of a basaloid squamous cell carcinoma was made. Further molecular studies were also done due to the complex presentation of the tumor. EWSR testing with break-apart analysis confirmed* EWSR1 *and* FLI1 *rearrangements. Further confirmation was done with RT-PCR. The case was found to be positive for EWS-FLI-1 translocation. The revised immunohistochemistry panel showed CD99, ERG, FLI1, and synaptophysin positivity. The lesion was reclassified as an adamantinoma-like ES. Our case reinforces the fact that a subset of EFTs can show histomorphologic and immunohistochemical features of aberrant epithelial differentiation. These cases are difficult to differentiate from usual epithelial malignancies which occur in this region. This diagnostic pitfall can be avoided by the inclusion of CD99 and/or FLI1 in the immunohistochemical assessment of any round cell malignancy at any anatomic location. A strong and diffuse CD99 positivity should prompt molecular testing for the presence of* EWSR1 *gene rearrangements.

## 1. Introduction

Ewing's family of tumors are a very rare group of sarcomatous malignancies affecting the bone and soft tissues. Common to these tumors and their variants is the molecular abnormality (11;22) (q24;q12), which involves the* EWSR1 *and* FLI-1 *genes [1, 2]. They commonly affect pediatric and young adults. Approximately 5% of Ewing's sarcoma (EWS) occurs in the head and neck and have been recently described in the sinonasal tract, parotid gland, thyroid gland, and orbit [2].

The classic monomorphic small blue round cell (SBRCT) appearance of EFTs overlaps with that of other tumors commonly occurring at the same sites, such as alveolar rhabdomyosarcoma, olfactory neuroblastoma, NUT midline carcinoma, lymphoma, melanoma, and others [3].

The adamantinoma variant of EFTs exhibits histomorphologic and/or immunophenotypic evidence of squamoid differentiation. The histologic appearance of this morphologic variant commonly overlaps with squamous cell carcinomas of the head and neck region. It can also have a complex immunoprofile, characterized by diffuse reactivity to HMWCKs, CK5/6, p40, and p63 [4].

Because of these characteristics, diagnosis of EFTs and their morphologic variants always relies on a constellation of features, including morphology, immunohistochemistry (such as CD99 and FLI-1 positivity), and characteristic molecular abnormalities [2]. To date, three independent cases of adamantinoma-like EFTs have been reported in head and neck sites, including the soft tissue of the neck, parotid gland, and thyroid gland [1–7]. In this report, a case of sinonasal adamantinoma-like EFT with complex epithelial differentiation is described.

## 2. Case Report

The patient was an 18-year-old male who presented with a nasal mass. Preoperative imaging studies suggested a vascular lesion, and the patient underwent an incomplete excision of the mass. Initial histopathological findings documented a sinonasal basaloid squamous cell carcinoma, and subsequently, the patient underwent endoscopic craniofacial resection and reconstruction. Because of the complex tumor presentation, the tumor sample was sent for FISH cytogenetics: ESW-FLI-1 fusion analysis.

## 3. Histopathology and Immunohistochemistry

**Figure 1:**
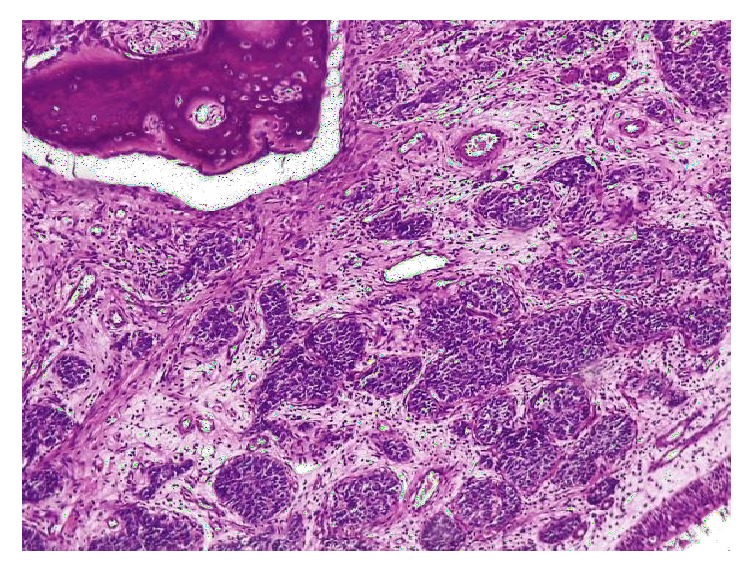
Adamantinoma-like EWS: ill-defined lesion composed of cells arranged in lobules and nests (H and E x 100).

**Figure 2:**
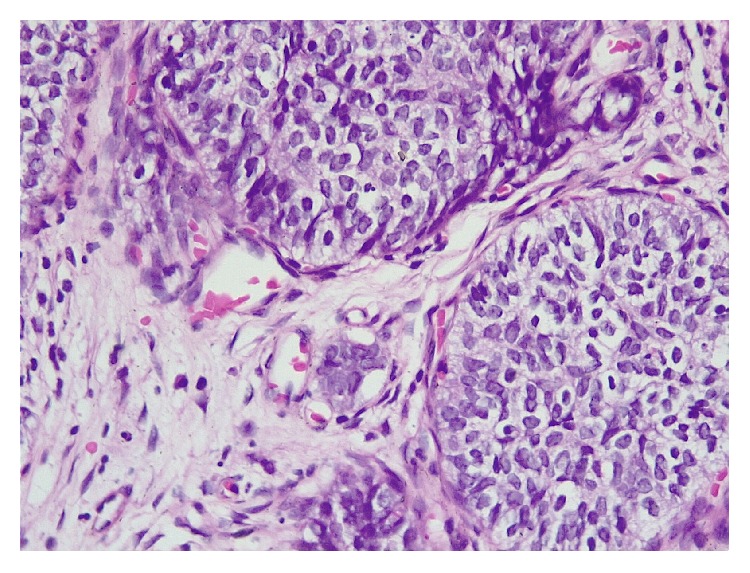
Adamantinoma-like EWS: H&E x400.

**Figure 3:**
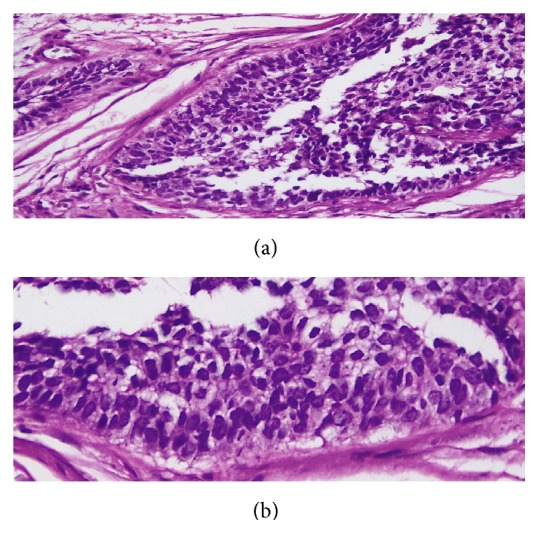
Adamantinoma-like EWS: prominent squamoid morphology with peripheral palisading. ((a) H&E X 200; (b) H&E X 400).

**Figure 4:**
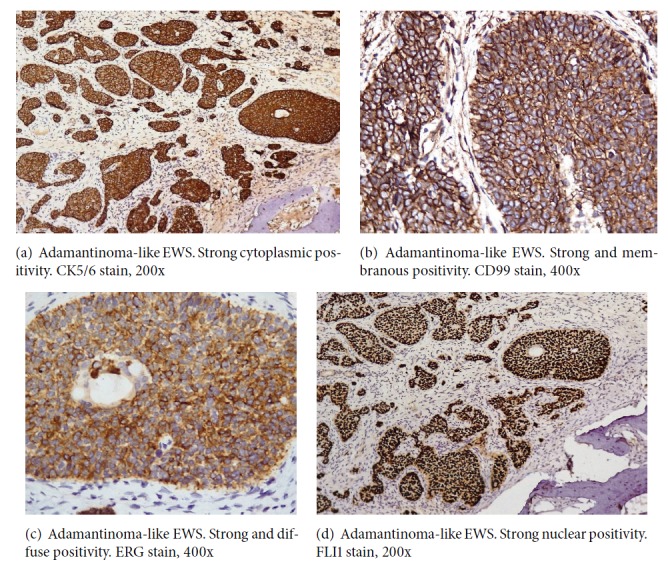


**Figure 5:**
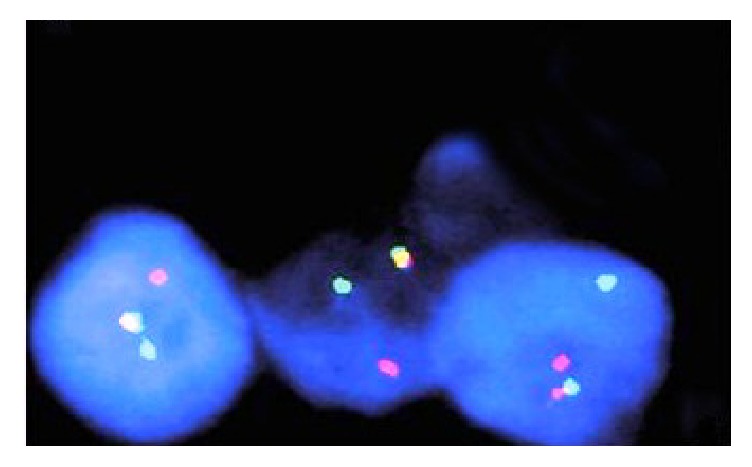
FISH image showing separation of orange and green fluorescence indicating presence of EWSR1 translocation.

Histological findings revealed a cellular malignant neoplasm composed of fairly monomorphic cells with focal squamoid and basaloid morphology arranged in lobules. The cells had vesicular nucleus, small nucleolus, and scanty cytoplasm with high mitotic activity. Peripheral palisading was observed. The surrounding stroma was fibrotic. Initial immunohistochemical panel was positive for PAN- CK, HMWCK, CK5/6, p40, and p63. The case was sent for molecular study because of the complex tumor presentation. EWSR testing with break-apart analysis confirmed* EWSR1 *and* FLI-1* rearrangements (Figure 5). Additional immunohistochemical analysis revealed strong, diffuse, membranous CD99, ERG, and FLI1 positivity, with focal dot-like positivity for synaptophysin. Immunoreactivity for p16, WT1, chromogranin, S100, EMA, vimentin, and desmin were negative in the lesional cells (Figures 1, 2, 3, and 4). These findings suggested that the tumor was initially misdiagnosed as a basaloid squamous cell carcinoma. Unlike previous reports of sinonasal EFT, strong and diffuse positivity was observed for HMWCK and P63 in this case.

Immunohistochemistry (IHC) pattern highlighted a complex epithelial differentiation, which was unique to this case. The diagnosis was revised to adamantinoma-like EWS with complex epithelial differentiation.

## 4. Materials and Methods

Tissue sections were collected from the sinonasal mass. Sections were fixed in 10% neutral buffered formalin and then subjected to routine processing. Four-*μ*m-thick sections were taken from the paraffin-embedded blocks and stained with hematoxylin (H) and eosin (E) stains. Sections were visualized using microscopy. Immunohistochemical analysis was performed using an automated immunostainer (Bond max Leica bio systems, USA) and automated LEICA detection system. Antigen retrieval was performed using bond refine polymer detection. A positive nuclear, cytoplasmic, and/or membranous expression in 10% or more of neoplastic cells qualified as “positive.” EWSR testing with break-apart analysis was performed at Oncquest laboratories, New Delhi, on formalin-fixed, paraffin-embedded tumor tissue using FISH probe-zytolight SPEC EWSR1 dual color break-apart probe. The test was developed, and its performance characteristics were determined by Oncquest lab consistent with NABL requirements. Reverse transcriptase PCR was performed at Neuberg Anand reference laboratories, Kochi, Kerala, India. Tumor tissue was identified and RNA was isolated using Trizol reagent in accordance with the manufacturer's recommendations.

### 4.1. Reverse Transcriptase PCR

**Figure 6:**
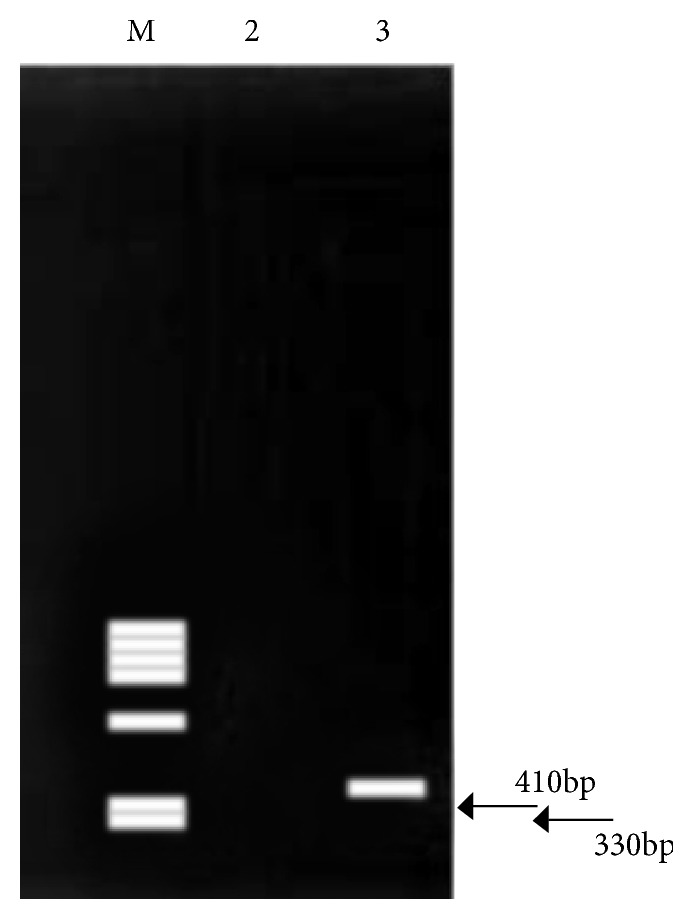
RT-PCR showing ESW-FLI1 fusion product.

RT-PCR was performed with ABL oligonucleotide primer. 30 cycles of RT-PCR were performed with each specific primer pairs (EWS 22x3 and FLI 1 11x3, EWS 22x8 and ERG 11), which amplified a 300 bp product. The EWS-FLI 1 product was either 330 bp (type 1 fusion) or 410 bp (type 2 fusion). Amplified PCR products were checked in a 2% agarose gel. One positive control t(11; 22) sample, one water only (no cDNA), negative control were included in each process. (Figure 6)

## 5. Discussion

In 1975, Van Haelst and de Hass Van Dorsser reported the first case of an adamantinoma-like ES with differential diagnosis between adamantinoma and atypical ES [8]. Subsequently, Moll et al. described CK8 and 18 positivity in the epithelial component in 1987 [9]. The presence of 11;22 translocation in these CK-immunoreactive cells was demonstrated by Bridge et al. in 1999, confirming these neoplasms as morphologic variants of ES. They named the lesion adamantinoma-like Ewing's sarcoma [10]. In 2005, Folpe et al. described EWS variants and demonstrated that HMWCK positivity was unique to a subset of EFTs, with all other keratin-positive and typical EFTs showing negative staining [11]. This was followed by several other studies describing similar unique subsets of EFTs. They termed this particular subset as adamantinoma-like EFT with complex epithelial differentiation [1, 4, 12].

## 6. EFT

Ewing's family of tumors are a group of sarcomatous malignancies that includes a spectrum of small blue round cell tumors (SBRCTs), such as osseous and extraosseous ES, peripheral neuroectodermal tumor (PNET), and Askin's tumor of the chest wall [3]. These tumors are considered to be derived from primitive pluripotent stem cells with the ability to differentiate into epithelial, mesenchymal, or neuronal lineages [13]. The corresponding phenotype derived from these lineages can express the three features to a varying extent. The term ES has been used to describe tumors that lack evidence of neuroectodermal differentiation, as assessed by light microscopy, immunohistochemistry, and electron microscopy. The term PNET, on the other hand, describes tumors with neuroectodermal features, as evaluated by one or more of these modalities [1].

Because EFTs have a common histogenesis, they share histopathological, immunocytochemical, and molecular features:

1. The histological features include sheets and a vaguely lobular growth pattern of monomorphic, uniform, small round cells. The cells display round nuclei containing fine chromatin, scanty clear or eosinophilic cytoplasm, and indistinct cytoplasmic membranes with a rich capillary network. The cytoplasm contains glycogen, which is detected using periodic acid Schiff staining and is diastase-degradable [14].
2. ES and PNET highly express* MIC2 *gene product, a30/32 kD surface antigen. The detection of this surface protein by CD99, although not specific, is very characteristic when there is strong membranous immunoreactivity in the majority of cells [15, 16]. Twenty to thirty percent of EFTs exhibit focal reactivity with low molecular weight CKs [11, 14]. Depending on the degree of neuroectodermal differentiation, tumor cells may also express neuron-specific enolase, synaptophysin, and S-100 protein [14]. Epithelial differentiation in EFTs shows positive staining for AE1/AE3 or CAM5.2. It ranges from 20% to 32% in either focal or diffuse pattern [10, 11, 17, 18]
3. These tumors are predominantly defined by* EWSR1 *rearrangements. Translocation (11;22) is specific to EFT family, although it has occasionally been reported in other tumor types. Confirmation of translocation t(11:22) (q24;q12) between the amino terminus of* EWSR1 *and the carboxy terminus of* FLI-1 *gene is present in nearly 90%–95% of ES/PNETs and has become an invaluable diagnostic marker [2–4, 10]. In 10%–15% of cases, the translocation t(21;12)(22;12), resulting in* EWS*-*ERG *(Ets-related gene) fusion, is observed.

Mutations can be detected by RT-PCR, FISH, and ISH.

In general, 9-20% of EFTs have a monomorphic SBRCT histomorphology. However, EFTs may present varying morphologies, including large cell ES, ES/PNET with hemangioendothelial features, synovial sarcoma-like ES, sclerosing ES, adamantinoma-like ES, or EFT with complex epithelial differentiation. Among these, recent reports of adamantinoma-like EWS have increased [4, 11].

## 7. Adamantinoma-Like EFT

Similar to classic EFT, head and neck adamantinoma-like EFT appears to generally affect young patients and may arise in a wide range of anatomic subsites, including periorbital soft tissues, thyroid gland, parotid gland, and even mucosal sites, such as the sinonasal tract [1–7]. Adamantinoma-like EFTs exhibit the common prototypical molecular integrity of 11;22 translocation and/or* EWS/FLI1 *or* EWS/ERG *fusion genes of typical EFTs. However, they have ultrastructural characteristics of both epithelial and neuroectodermal cells. Therefore, they are considered EFT with genotypic and phenotypic drift, exhibiting both epithelial and neuroectodermal differentiation.

Their neuroectodermal component displays a very monomorphic appearance with nuclear molding, salt and pepper chromatin. They show positive IHC for CD99, FLI-1, and synaptophysin, among others. A tendency toward neuroectodermal differentiation brings them close to the morphologic spectrum of other SBRCTs in the region. Immunohistochemistry usually helps clarification [3].

Their epithelial component is characterized by squamoid or basaloid morphology with squamous eddies, intercellular bridges, ducts, and glands. The epithelial component, which is typically partial, shows positive IHC for p63, p40, and CKs. A higher tendency toward true and complete epithelial differentiation brings them under the morphologic spectrum of other common epithelial malignancies. The resulting effect is a prominent squamoid morphology and super added cytokeratin immunoprofile, with focal/diffuse HMWCK positivity. This can strongly indicate a carcinoma, especially squamous cell carcinoma, a far more common malignancy of the head and neck. However, the proportion of epithelial and neuroectodermal differentiation varies between adamantinoma- like EFTs [3].

True and complex epithelial differentiation as a unique feature of adamantinoma-like EFTs was demonstrated by Folpe et al. in 2005. The authors showed diffuse positivity for HMWCK, CK5/6, and p63 in a subset of EFTs resembling squamous cell carcinoma. They strongly affirmed that HMWCK positivity was unique to a subset of EFTs, with all other keratin-positive and typical EFTs exhibiting negative staining [11]. The work by Weinreb et al. in 2008 and Kikuchi et al. in 2013 followed [7, 16, 17], describing similar unique subsets of EFT. These tumors exhibited undifferentiated round cells, basaloid pattern, stromal desmoplasia, and peripheral nuclear palisading, which are not typical of adamantinoma- like EFTs. The authors consolidated the histologic and immunohistochemical differences between their cases and typical adamantinoma-like EFT and, instead of using the term “adamantinoma-like,” they adopted the phrase “extraskeletal EFT with complex epithelial differentiation.”

In the present case, extensive squamoid morphology, basaloid pattern, peripheral nuclear palisading, and stromal desmoplasia were observed. The initial immunohistochemical panel was positive for PAN-CK, HMWCK, CK5/6, p40, and p63. The tumor was initially misdiagnosed as basaloid squamous cell carcinoma. Because of the complex presentation of the tumor, the case was sent for molecular evaluation. FISH was positive for the* EWS*-*FLI-1* fusion, confirming diagnosis of ES. Additional IHC studies revealed strong, diffuse, membranous CD99 with focal dot-like positivity for synaptophysin. Immunoreactivity for p16, WT1, chromogranin, S100, EMA, vimentin, and desmin were negative in the lesion cells. Unlike previous reports of sinonasal EFT, strong and diffuse positivity was observed for HMWCK. IHC pattern also highlighted a complex epithelial differentiation, unique to this case. The revised diagnosis issued was adamantinoma-like EWS with complex epithelial differentiation.

This case, together with those reported by Folpe et al. and Weinreb et al. are considered within the common spectrum of EFT with complex epithelial differentiation. Diffuse expression of HMWCK can indicate complex and true epithelial differentiation. The cause for this remains unclear, urging the need to investigate further cases of this variant to fully understand its place in the EFT family. An incorrect diagnosis of squamous cell carcinoma in these patients may result in radiation therapy of the head and neck structures, neck dissection, and tonsillectomy. The reclassification to EFT in such cases requires a change in treatment protocol.

## 8. Conclusion

A case of sinonasal adamantinoma-like EFT with complex epithelial differentiation was established and genetically confirmed in this study. We consider that the cases reported by Weinreb et al., Kikuchi et al., and Fuji et al., together with the presently described case, are within the common spectrum of EFT with complex epithelial differentiation. The histological features suggest that the adamantinoma-like and complex epithelial subtypes are within a common tumor spectrum.

Precise tumor classification is crucial for establishing prognosis and guiding appropriate therapeutic strategies. Diffuse p40/p63 immunostaining in combination with synaptophysin positivity should prompt suspicion of adamantinoma-like EFT. Further inclusion of CD99 in undifferentiated SBRCTs of the head and neck can help circumvent all the above pitfalls. CD99 immunoreactivity should prompt consideration for molecular studies, including the analysis of both EWSR1 and FLI-1 even in the presence of strong cytokeratin expression or focal keratinization. This will help identify cytokeratin-positive EFTs with a very aggressive behavior and precisely classify them as adamantinoma-like EFTs [19].

## Data Availability

The data used to support the findings of this study are available from the corresponding author upon request.
